# Meckel’s Diverticulitis in a Teenager With Unknown Intestinal Malrotation: A Case Report and Review of the Literature

**DOI:** 10.7759/cureus.23846

**Published:** 2022-04-05

**Authors:** Elissavet Symeonidou, Konstantinos Kiroplastis, Maria S SidiropouIou, Ioannis Gkoutziotis, Apostolos Kamparoudis

**Affiliations:** 1 Surgery, Ippokrateio General Hospital, Thessaloniki, GRC; 2 Radiology, Ippokrateio General Hospital, Thessaloniki, GRC

**Keywords:** acute abdomen, congenital abnormalities, midgut malrotation, intestinal malrotation, meckel’s diverticulum

## Abstract

The clinical presentation of congenital abnormalities in adult life is a rare condition since they usually make their appearance in early childhood. A combination of two different congenital deformities is even more infrequent, a fact that might complicate the differential diagnosis of acute abdomen. This is a case report of an inflamed Meckel’s diverticulum in a 16-year-old male with intestinal malrotation presented in an acute setting, and a review of the literature. The patient presented at the emergency department with an atypical abdominal pain located in the right abdomen and quite elevated inflammatory markers. Computed tomography revealed Meckel’s diverticulitis in combination with intestinal malrotation, findings that were confirmed intraoperatively. A partial enterectomy with a side-to-side anastomosis was performed, and the patient was discharged uneventfully. Only a few cases of this combination have been reported in the literature till nowadays. This article indicates the importance of the computed tomography scan in the differential diagnosis of abdominal pain since it might reveal rare clinical entities and determine the further therapeutic plan. Furthermore, it is a reminder that congenital abnormalities might make their clinical appearance not only in early childhood but also in adult life, pointing out the ability of the general surgeon to deal with such cases.

## Introduction

Congenital abnormalities of the gastrointestinal tract, such as Meckel’s diverticulum and intestinal malrotation, are rare clinical entities, and they usually present clinically in early childhood. Meckel’s diverticulum appears in a prevalence ranging between 0.3% and 2.9% of the population [1], but only 3% of the cases become symptomatic [2]. Likewise, the incidence of intestinal malrotation is estimated at about 1 in 6000 births [3], though the real percentage is higher since many individuals remain asymptomatic. The coexistence of different congenital abnormalities is also possible. Because of the rarity of this clinical condition, the differential diagnosis of abdominal pain may become complicated and even delayed. The computed tomography scan nowadays is a useful and widely available diagnostic tool that determines further therapeutic management [4].

## Case presentation

A 16-year-old Caucasian male presented to the emergency department with a history of seven-day right abdominal pain, progressively deteriorating, and coexisting anorexia. No history of previous abdominal surgery or any health problems from his medical history were noted.

His vital signs were stable; the blood pressure was 125/72 mm Hg with 80 heartbeats per minute, 100% oxygen saturation, and a temperature of 37.2 degrees Celsius. The clinical examination revealed tenderness in the right lower, as well as upper, abdominal quadrant. Blood tests revealed elevated inflammatory markers, white blood cells were 12000 x 10^3 /μL (3800-10500x10^3), with 74% neutrophils, and C-reactive protein was 17,4 mg/L (>6 mg/L).

**Figure 1:**
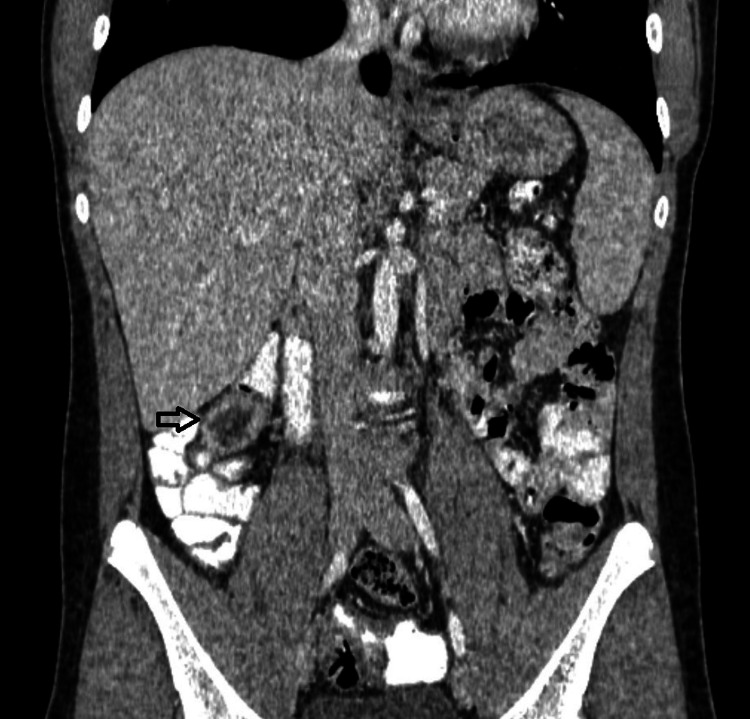
Enhanced CT with oral contrast, coronary view showing a tubular formation located beneath the liver, enhancing through the arterial phase, suggesting an inflamed Meckel’s diverticulum

**Figure 2:**
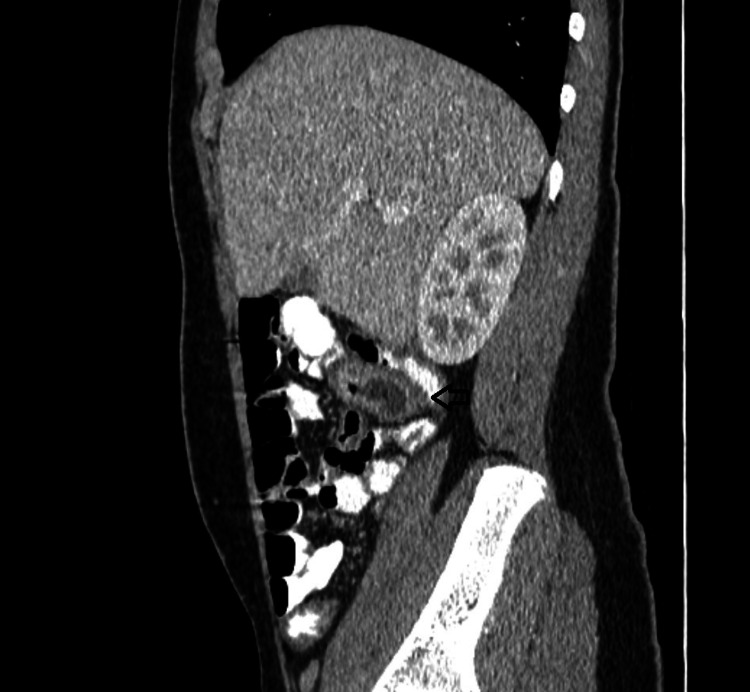
Enhanced CT with oral contrast, sagittal view showing the communication of Meckel’s diverticulum with the small intestine

**Figure 3:**
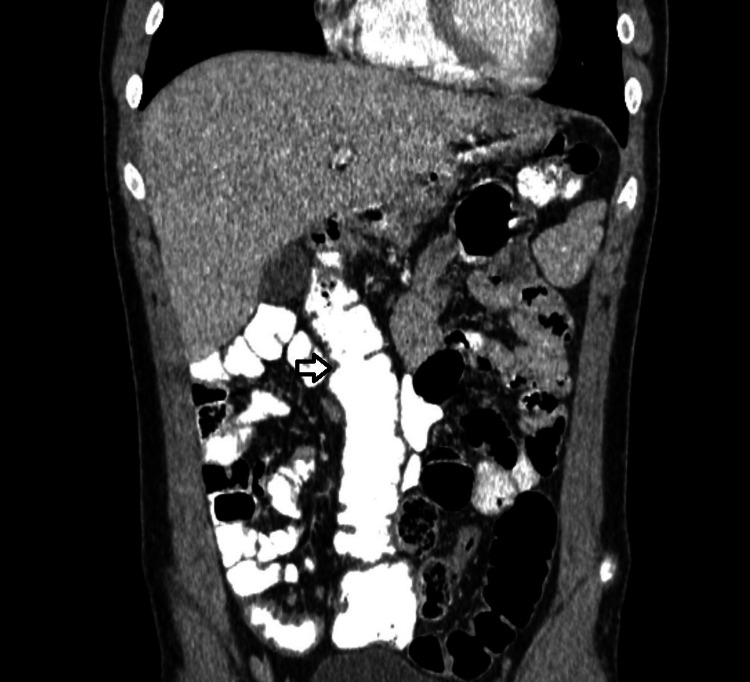
Enhanced CT with oral contrast, coronary view showing the ascending colon (white arrow) lying in the midline and the small intestine, located at the right side of the abdomen

**Figure 4:**
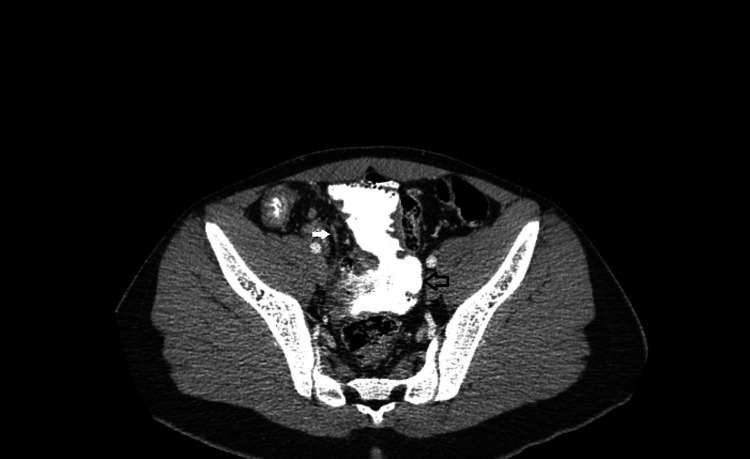
Enhanced CT with oral contrast, axial view showing the appendix (white arrow) and the cecum

**Figure 5:**
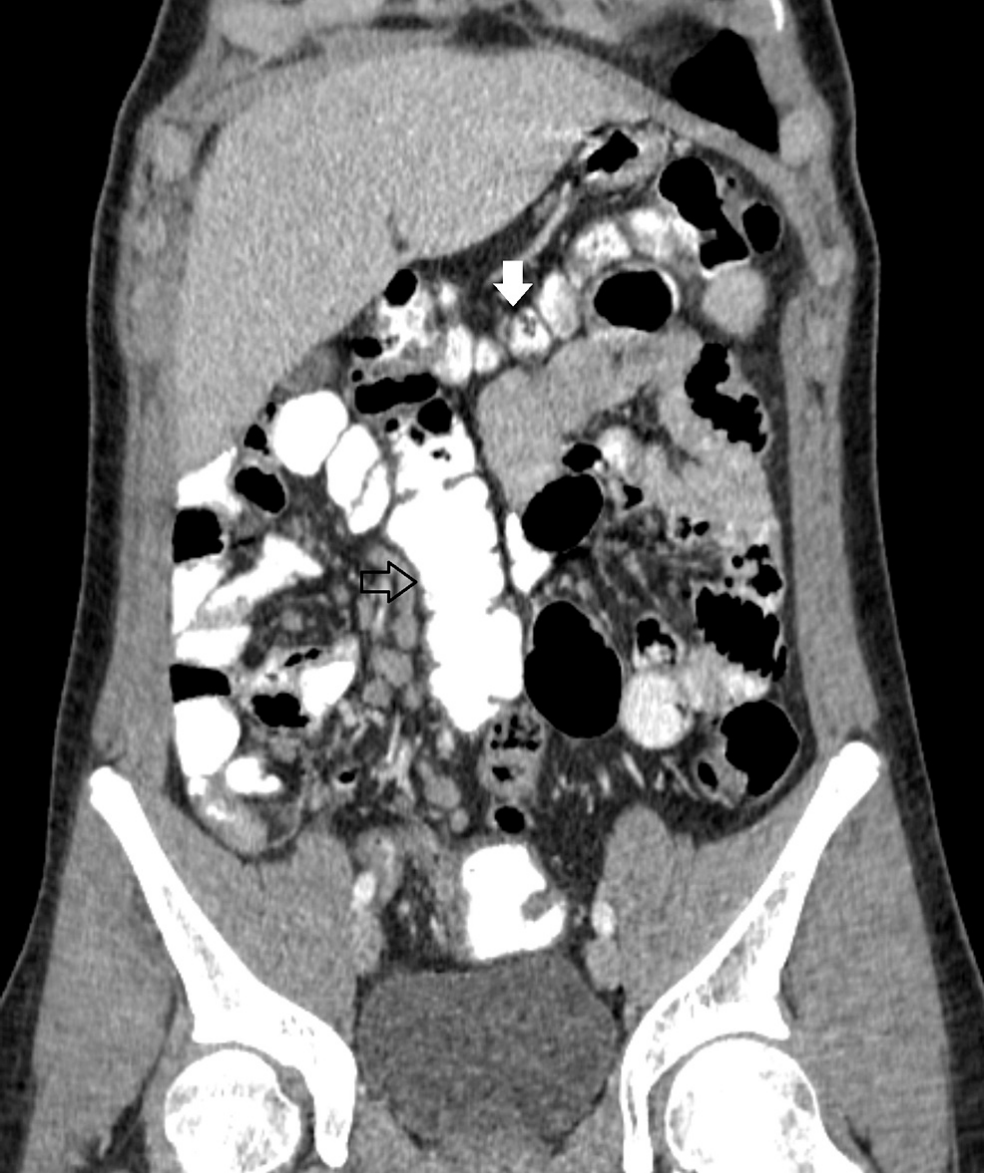
Enhanced CT with oral contrast showing the transverse colon (white arrow) lying lateral to the ascending (blue arrow) towards the left abdomen

Ultrasound of the right lower abdomen was inconclusive. Investigation was decided to proceed with a computed tomography scan, in which a tubular formation, 5,1x2,6x2,3 cm in size, was described beneath the liver, containing air and liquid, enhancing during the arterial phase and connected to the small intestine, suggesting an inflamed Meckel’s diverticulum (Figures 1, 2). Moreover, the cecum, the ascending colon (Figure 3), and the appendix (Figure 4) were located at the midline, whereas the small intestine was lying lateral to the ascending colon (Figure 5), suggesting intestinal malrotation.

**Figure 6:**
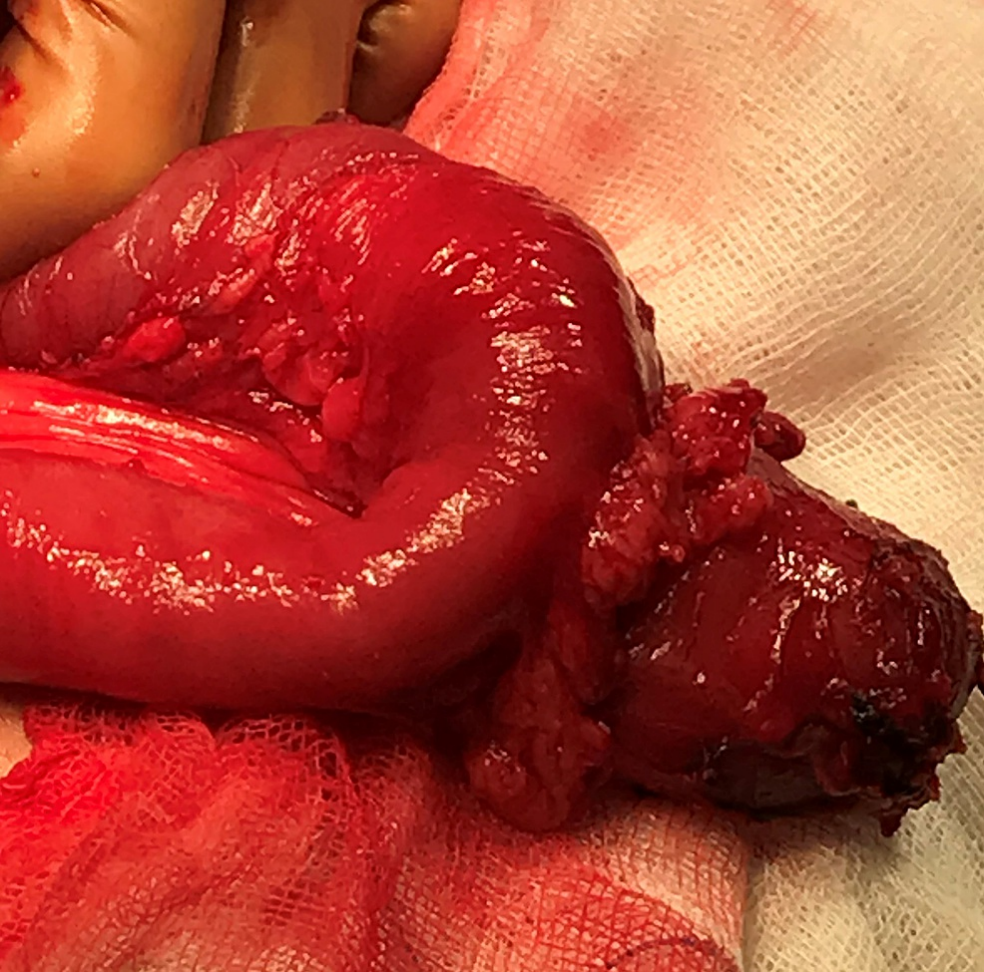
Intraoperative image of the inflamed Meckel’s diverticulum

The patient underwent an exploratory laparotomy, where through a midline incision, the inflamed Meckel’s diverticulum (Figure 6) was recognized above the duodenum and beneath the right hepatic flexure after the division of peritoneal bands. It was located within 2 feet of the ileocecal valve. A partial enterectomy with a stapled side-to-side anastomosis was performed. The small intestine was repositioned to the right side of the abdomen, whereas the ascending colon and cecum to the midline. The laparoscopic approach was not considered a safe option based on the atypical anatomic location of the inflamed Meckel’s diverticulum and the lack of experience of our center with the management of congenital abnormalities.

**Figure 7:**
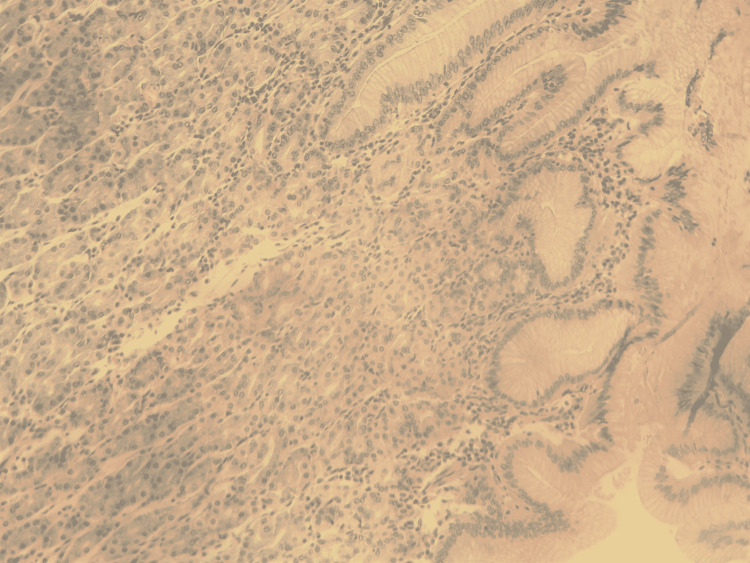
Histopathology with Hematoxylin and Eosin staining x100, showing inflamed Meckel’s diverticulum containing ectopic gastric mucosa

The histopathology report confirmed the diagnosis of an inflamed Meckel’s diverticulum, 4 cm in size, containing ectopic gastric tissue (Figure 7). 

The postoperative period was uneventful, and the patient was discharged on the fourth postoperative day. During a follow-up period of twelve months, the patient presented no recurrence of the abdominal pain or any other pathological symptoms.

## Discussion

Meckel’s diverticulum is the most common anatomic abnormality of the gastrointestinal tract, with a prevalence ranging between 0.3 and 2.9% [1], according to a recent systematic review published by Hansen and Soreide. It occurs as the result of incomplete erasure of the omphalomesenteric duct during embryogenesis. There is a clear male predominance (1.5-4 males for every female) [1], and it is considered a disease of the youth, as more than 50% of the patients who become symptomatic are under the age of 10. Approximately 3% of the individuals will become symptomatic and eventually be surgically treated [2].

Τhe most common clinical manifestations of Meckel’s diverticulum are bleeding because of ectopic gastric or pancreatic tissue, inflammation (diverticulitis) which can evolve into perforation and therefore peritonitis, and obstruction by the mechanism of intussusception or volvulus, when Ladd’s bands are present. Meckel’s diverticulum might also present as an inguinal hernia (Littre’s hernia) in rare cases. The existence of benign or malignant tumors is infrequent but also possible.

The midgut formation takes place between the fourth and 10th weeks of the embryonic period. At first, the small bowel rotates 90 degrees counterclockwise around the superior mesentery artery axis. Then the small bowel elongates and rotates again 180 degrees counterclockwise, taking its final position in the peritoneal cavity. The incidence of symptomatic intestinal malrotation is estimated to be 1 in 6000 births [3]. 90% of the cases with intestinal malrotation present within the first year of life, whereas the incidence in adults is estimated at around 0.2%, with most of the cases presenting in the acute setting [5]. Intestinal malrotation is categorized as the non-rotation type, the reverse rotation type, the incomplete rotation, and the anomalous fixation of the mesentery type. The most common type is non-rotation, as described in the case presented.

The combination of Meckel’s diverticulum and intestinal malrotation is a rare clinical entity, with only a few case reports published in the literature. A literature search using the terms Meckel’s diverticulum and intestinal malrotation or midgut malrotation was conducted using the PubMed database. Based on this search, only 10 case reports were noted, with nine being in the English language and therefore included in this review (Table 1).

**Table 1:** Case reports of Meckel’s diverticulum and intestinal malrotation M - man, F - female, MD - Meckel’s diverticulum, CT - computed tomography, NM - not mentioned, GI - gastrointestinal, PET - positron emission tomography, MT - magnetic Tomography

Source	Age	Sex	Symptoms	Clinical examination	Imaging	Intraoperative findings	Treatment	Other congenital abnormaiitites	Histology report	Presence of chronic abdominal symptoms
Harvey et al. [6]	47 years old	F	Periumbilical pain, vomiting	Midline mass	X-rays, barium meal, barium enema	Inflammation	Excision of terminal ileum and cecum together with MD and end-to-end anastomosis	-	Gastric like mucosa in the MD	yes
Harvey et al. [6]	17 years old	M	Colicky central abdominal pain	No distension or palpable mass	X-rays, barium studies	Obstruction of ileum	Removal of MD, adhesiolysis	-	Normal small bowel mucosa	yes
Burgard et al. [7]	65 years old	M	Increasing abdominal pain, fever, nausea	Periumbilical and left lower quadrant tenderness	CT	Inflamed MD containing calculus	Exploratory laparoscopy, segmental intestinal resection, occasional appendectomy	-	Inflammed gastric mucosa, perforation	NM
Taylor et al. [8]	8 years old	M	Periumbilical pain, nausea, vomiting	Erythematous tender paraumbilical mass	NM	Small bowel volvulus on a shortens mesentery, abscess	V-configuration resection of MD, appendectomy, adhesiolysis, suction of the abscess, and excision of necrotic tissue	-	Perforated, gangrenous MD	yes
Mushtaq et al. [9]	19 months old	M	Malaise, vomiting	Abdominal distension	X-rays, US, CT	Small bowel obstruction	Resection of MD and ischaemic terminal ileum, Ladd’s procedure, appendicectomy	Left-sided superior vena cava	NM	no
Lee et al. [10]	19 years old	F	Left lower abdomen pain	NM	Sonography, CT, MT, PET, colonoscopy	abscess	Laparoscopic Ladd’s procedure and segmental resection of the ileum	-	Adenocarcinoma from MD, T4N1M0, stage IIIa	NM
Elrouby et al. [12]	Neonate	M	Gastroschisis	Herniation - gastroschisis	--	-	Ladd’s procedure, excision of the duplicated lesion, but not MD	Dublication of the small bowel		-
Mirza et al. [13]	18 months old	M	Fever, cough, respiratory distress, vomiting	Nasal flaring, subcostal and intercostal retractions, course crepts	Chest X-ray, CT of the chest	Hiatus hernia, Ladd’s band, pulmonary sequestration	Ladd’s procedure, resection of pulmonary sequestration and MD, repair of hiatus hernia	Hypospadias, hiatus hernia, pulmonary sequestration	Heterotrophic mucosa	yes
Weitzman et al. [16]	Neonate	F	Respiratory distress, vomiting	NM	Upper GI series, aortogram	Partial intestinal obstruction	Right thoracotomy, resection of bronchogastric fistula, MD and pulmonary sequestration, hiatal hernia repair, Thal fundoplication, Ladd procedure	Pulmonary sequestration, bronchogastric fistula, esophageal hiatal hernia	NM	-
Weitzman et al. [16]	16 months old	F	Chronic cough, recurrent pneumonia,		X-rays, upper GI series, aortogram	Long-standing small bowel obstruction	Right thoracotomy, right lower lobectomy, division of bronchogastgric fistula, vagotomy, hiatal hernia repair, Thal fundoplication, pyloroplasty, Ladd procedure, partial enterectomy including MD	Pulmonary sequestration, bronchogastric fistula, esophageal hiatal hernia	No gastric mucosa include	-
Basani et al. [15]	3 months old	M	Fever, vomiting, rapid breathing	Decreased air entry on the left side	X-rays, CT	Herniation of small bowel, colon, and spleen into left hemithorax	Left subcostal incision, hernia repair, Ladd’s procedure, appendectomy, intestinal repositioning, excision of MD	Congenital diaphragmatic hernia	NM	-

Harvey and Giles first reported two clinical cases of this association in adults in 1974 [6]. The age of clinical presentation ranges from infancy to elderly [7], and it involves complications, such as perforation [7, 8], obstruction [9], and even adenocarcinoma [10]. A common symptom among many cases is the presence of chronic pain before the acute onset and the absence of Ladd’s bands, explaining the older age of appearance [8]. All of the cases published in the literature are presented in Table 1. Interestingly, in our case, the patient was 16 years old with no history of previous abdominal pain until Meckel diverticulitis presented.

Ford et al [11] announced that in up to 11% of the children with intestinal malrotation, Meckel’s diverticulum is identified. However, the accurate percentage remains unknown since many individuals remain asymptomatic.

Several combinations of congenital deformities, including Meckel’s diverticulum and midgut malrotation, have been reported. Elrouby et al. [12] presented a case of a neonate with gastroschisis, malrotation, midgut duplication, and Meckel’s diverticulum. Bilal Mirza et al. [13] announced a case of an 18-year-old male with a symptomatic pulmonary sequestration cyst and diaphragmatic hernia, where malrotation with Ladd’s band and Meckel’s diverticulum were also present. It is of interest that malrotation is seen in 42% of the patients with congenital diaphragmatic hernia [14]. Basani et al. presented a case of a three-month-old infant with a diaphragmatic hernia, Meckel’s diverticulum, and midgut malrotation [15]. Weitzman and Brennan described two cases with bronchogastric fistula, pulmonary sequestration, malrotation, and Meckel’s diverticulum [16]. Although the exact pathogenesis of this association remains unknown, the diagnosis is set in early childhood when multiple congenital abnormalities are present.

There is a variety of diagnostic imaging tools available for the evaluation of the complications of Meckel’s diverticulum. A plain radiograph might reveal small bowel obstruction, an enterolith, and in rare cases, a calcified Meckel’s diverticulum. Ultrasonography is a widely available tool, frequently used for the initial diagnostic management of acute abdomen in children because of the lack of radiation exposure, but its sensitivity is low, and its findings depend on the operator’s experience. Computed tomography scan, with thin-slice images and the capability of multiplanar reconstruction, is the imaging of choice for the complications of Meckel diverticulum since it has higher sensitivity, especially in the diagnosis of small bowel obstruction. Only in cases of gastrointestinal bleeding from ectopic gastric mucosa, in pediatric patients, 99m-Tc scintigraphy might be proven superior [17]. However, the preoperative diagnosis of Meckel’s diverticulum is achieved only in 5-15% of the patients, and therefore most of the cases are diagnosed during laparoscopy, especially in the absence of fecolith or foreign body [7]. In the presented case, the evaluation began with ultrasonography, which was inconclusive, and continued with computed tomography scan, which set the accurate diagnosis and was determinant for the further therapeutic management of the patient. If the computed tomography scan was inconclusive or unavailable, exploratory laparoscopy would be an appropriate next step.

Ladd’s procedure, laparoscopic or open, is the standard surgical operation for malrotation with or without intestinal volvulus. It involves the division of peritoneal bands, Ladd’s bands or other adhesions, widening of the mesentery base, the repositioning of the small intestine and ascending colon to the right and left side of the abdomen, respectively, as well as appendicectomy, with the last one not being mandatory in adults. It is the treatment of choice in case of acute obstruction, chronic abdominal pain, or non-specific symptoms due to malrotation. If intestinal malrotation is incidentally discovered during a laparotomy, Ladd’s procedure is also recommended in order to avoid future volvulus [18].

A reasonable question that arises is whether or not to perform incidental appendectomy in case of malrotation in adults. The presence of malrotation suggests a problem for the differential diagnosis in case of a possible future abdominal pain since the appendix is not located at its typical anatomic place. Taking into consideration that the lifetime risk for appendicitis is approximately 7% [19] and the complication rate is extremely low, incidental appendectomy is strongly recommended in case of a laparotomy for any other pathology in cases with malrotation as a cost-saving procedure that would prevent future radiation and even surgery. On the other hand, according to a study published by Brungardt et al. [20], adults with malrotation who underwent simultaneous appendicectomy were more likely to have a clean-contaminated wound classification whether those who did not undergo appendicectomy were more likely to have a clean wound classification. However, no significant differences in the postoperative outcomes were noticed. Since it was not really necessary, we decided not to perform an appendicectomy.

Moreover, the management of an incidentally found Meckel’s diverticulum still remains controversial. The lifetime risk of developing complications is up to 3% [2], and it increases with younger age, the presence of ectopic gastric tissue or fibrous bands, or when the length of the diverticulum is more than 2 cm [1]. These factors should be taken into consideration by the surgeon. In the aforementioned case, taking into consideration diverticulum size and the level of its inflammation, a partial enterectomy was performed, followed by a side-to-side stapled anastomosis.

In conclusion, this case report is a reminder that, although rare, it is possible that congenital abnormalities make their presentation not only later in life but also in the acute setting. In these cases, clinical awareness is essential for the diagnosis and the proper therapeutic management. Atypical abdominal pain, especially when combined with chronic symptoms, raises clinical suspicion, and a computed tomography scan is the imaging of choice. Another important take-away lesson from this case is that every general surgeon should be familiar with Ladd’s procedure and the indications of a simultaneous appendicectomy, as well as the resection of an inflamed or not Meckel’s diverticulum.

## Conclusions

The combination of midgut malrotation and Meckel’s diverticulum is a rare clinical entity presenting in adult life, which is usually associated with chronic symptoms before the acute onset. Their coexistence with other congenital abnormalities has also been reported in the literature. Increased awareness of these anatomic variants is essential among emergency physicians, radiologists, and surgeons in cases of atypical abdominal pain, and further imaging is recommended before proceeding with surgery. The computed tomography scan is a necessary tool in terms of diagnosis and further therapeutic management of the acute abdomen. Every general surgeon should be capable of performing a Ladd’s procedure; otherwise, the assistance of a pediatric surgeon is a reasonable option. Incidental appendectomy is recommended in patients with malrotation in order to avoid future diagnostic dilemmas.

## References

[1] Hansen CC, Søreide K (2018). Systematic review of epidemiology, presentation, and management of Meckel's diverticulum in the 21st century. Medicine (Baltimore).

[2] Lindeman RJ, Søreide K (2020). The many faces of Meckel's diverticulum: update on management in incidental and symptomatic patients. Curr Gastroenterol Rep.

[3] Hartman GE (2015). Intestinal obstruction. Neonatology: clinical practice and procedures.

[4] Kotha VK, Khandelwal A, Saboo SS, Shanbhogue AK, Virmani V, Marginean EC, Menias CO (2014). Radiologist's perspective for the Meckel's diverticulum and its complications. Br J Radiol.

[5] Emanuwa OF, Ayantunde AA, Davies TW (2011). Midgut malrotation first presenting as acute bowel obstruction in adulthood: a case report and literature review. World J Emerg Surg.

[6] Harvey JS, Giles GR (1974). Associated midgut malrotation and Meckel's diverticulum presenting in adult life. Br J Surg.

[7] Burgard M, Cherbanyk F, Pugin F, Egger B (2021). Perforated Meckel's diverticulitis in a patient with unknown intestinal malrotation: clinical pitfall. Case Rep Surg.

[8] Taylor H, Venza M, Badvie S (2015). Concurrent perforated Meckel's diverticulum and intestinal malrotation in an 8-year-old boy. BMJ Case Rep.

[9] Mushtaq N, Elwood E, Westwood E, Macdonald A, Saxena AK, Bretherton J (2022). Intestinal malrotation and Meckel's diverticulitis in a 19-month-old boy. BJR Case Rep.

[10] Lee JK, Kwag SJ, Oh ST, Kim JG, Kang WK (2013). Adenocarcinoma arising from Meckel's diverticulum in the ileum with malrotation of the midgut. J Korean Surg Soc.

[11] Ford PV, Bartold SP, Fink-Bennet DM, Jolles PR, Lull RJ, Maurer AH, Seabold JE (1999). Procedure guideline for gastrointestinal bleeding and Meckel’s diverticulum scintigraphy. J Nucl Med.

[12] Elrouby A, Maher A (2021). Gastroschisis with malrotation, gut duplication and Meckel's diverticulum; a rare association. J Pediatr Surg Case Rep.

[13] Mirza B, Saleem M, Ijaz L, Qureshi A, Sheikh A (2011). Pulmonary sequestration cyst in a patient of cerebral palsy. Lung India.

[14] Schropp KP, Garey CL (2010). Meckel’s diverticulum. Ashcraft’s pediatric surgery, 5th ed.

[15] Basani L, Aepala R, Reddy BM (2016). Congenital diaphragmatic hernia, Meckel's diverticulum and malrotation in a 3-month-old infant. Afr J Paediatr Surg.

[16] Weitzman J, Brennan P (1998). Bronchogastric fistula, pulmonary sequestration, malrotation of the intestine, and Meckel’s diverticulum - a new association. Journal of Pediatric Surgery.

[17] Ichikawa S, Onishi H, Motosugi U (2020). Imaging findings of acute abdomen due to complications of Meckel diverticulum. Can Assoc Radiol J.

[18] Panda N, Bansal NK, Narasimhan M, Ardhanari R (2014). Laparoscopic correction of intestinal malrotation in adult. J Minim Access Surg.

[19] Bhangu A, Soreide K, Di Saverio S, Assarsson JH, Drake FT (2015). Acute appendicitis: modern understanding of pathogenesis, diagnosis, and management. Lancet.

[20] Brungardt JG, Liebscher SC, Schropp KP (2021). Malrotation correction in the adult population. World J Surg.

